# Supplementary material for: Method for predicting dynamic current-carrying capacity of transmission lines by integrating improved VMD and time-varying ensemble model
Source: PLoS One. 2026 Feb 17;21(2):e0342293. doi: 10.1371/journal.pone.0342293 (PMC12912545; doi:10.1371/journal.pone.0342293)
Supplement: Attachment — Submitted filename: Response to reviewers.docx [file pone.0342293.s006.docx]

**Response to Reviewers' Comments**

**# Reviewer 1**

**Question 1:**

In this study, SMA was adopted for VMD hyperparameter optimization. Could the author provide theoretical and experimental analyses with other advanced optimization algorithms (such as particle swarm optimization and gray Wolf optimizer) to more reliably prove the rationality of choosing SMA, especially in terms of convergence speed and avoiding local optimality in the context of DTR data?

**Reply to Question 1:**

We sincerely appreciate the reviewer for sparing valuable time to conduct a meticulous review of the manuscript. Your comments have accurately identified the key aspects requiring improvement in the research, providing crucial guidance for enhancing the academic depth and engineering practicality of the paper. We have carefully studied the review comments and formulated a detailed revision plan in conjunction with the original content. The specific responses are as follows:

To fully demonstrate the superiority of the Slime Mould Algorithm (SMA) in the Demand Response Time (DTR) data scenario, we have supplemented the original manuscript from two aspects: comparison of theoretical characteristics and extended experimental verification:

**1. Theoretical Level**

**1.1 Adaptability of Convergence Speed**

Firstly, the core characteristics of DTR data are strong non-stationarity and multi-scale time-variability. Specifically, DTR values are influenced by the coupling of long-term periodic changes in solar radiation and ambient temperature, as well as short-term random disturbances in wind speed and direction, leading to dynamic migration of data distribution over time. Furthermore, this non-stationary characteristic directly triggers dynamic changes in the Variational Mode Decomposition (VMD) hyperparameter space (number of decomposition components *K* and penalty factor α). For example, during periods of high temperature and strong sunlight in summer, DTR exhibits significant high-frequency fluctuations, and the optimal hyperparameters need to be biased towards "narrow bandwidth and high penalty" to avoid over-decomposition; in contrast, during stable low-temperature periods in winter, the optimal hyperparameters should shift to "wide bandwidth and low penalty" to retain trend features. This imposes dual requirements on the hyperparameter optimization algorithm: it must quickly adapt to dynamic changes in the hyperparameter space while flexibly switching between "global exploration (covering a wide range of parameters) and local exploitation (locking in the optimal solution)", avoiding degradation in decomposition performance caused by mismatches between the optimization rhythm and data characteristics.

Next, we analyze the principle differences between the SMA, Particle Swarm Optimization (PSO), and Grey Wolf Optimizer (GWO) algorithms in detail to theoretically prove the superiority of SMA in the DTR data scenario.

(1) The primary limitation of the PSO algorithm lies in its convergence speed, which mainly depends on an artificially preset inertia weight decay strategy. This strategy is inherently "time-driven" rather than "data-driven", making it unable to respond to dynamic changes in the DTR data hyperparameter space. If the preset inertia weight decays rapidly, when DTR data suddenly exhibits high-frequency fluctuations (e.g., sudden gusts) leading to an expansion of the hyperparameter space, PSO has already entered the local exploitation phase. Insufficient exploration makes it difficult to cover the new optimal parameter interval, resulting in reduced decomposition performance. Additionally, if the preset inertia weight decays slowly, when DTR data enters a stable period (e.g., low-temperature nighttime environments) and the hyperparameter space tends to stabilize, PSO remains in the global exploration phase, leading to convergence delays and failure to lock in the optimal solution in a timely manner.

(2) The GWO algorithm achieves convergence through a hierarchical update mechanism involving α (leader), β (follower), and δ (explorer). The switch between global exploration and local exploitation is determined by the fixed assignment of hierarchical roles, lacking flexibility. Specifically, throughout the iteration process, α always dominates local exploitation (guiding the population to move towards the optimal position), while β and δ only assist in searching. This fixed hierarchical relationship results in the algorithm’s slow response to dynamic changes in the DTR data hyperparameter space. For example, when DTR data switches from high-frequency fluctuations in summer to stable trends in winter, the hyperparameter space transitions from "wide bandwidth and low penalty" to "narrow bandwidth and high penalty", but the α hierarchy of GWO still maintains the local exploitation mode, failing to quickly expand the search range. Furthermore, GWO’s convergence speed relies on the encircling behavior of wolf pack individuals, and its exploration capability is determined by the random values of coefficients, lacking a dynamic adjustment mechanism related to the fitness of DTR data. When the hyperparameter space contracts or expands due to data non-stationarity, it is difficult to adjust the ratio of exploration to exploitation, leading to convergence delays.

(3) Through the synergistic coupling of the dynamic weight coefficient *W* and control parameter *p*, SMA constructs an optimization logic that accurately matches the characteristics of DTR data. In the early stages of iteration, the non-stationarity of DTR data leads to uncertainty in the range of the hyperparameter space, requiring the algorithm to prioritize global exploration to cover potential optimal parameter intervals. At this point, the control parameter *p* is calculated using Equation 7 in the original manuscript. Due to the large difference in fitness among the initial population, the value of *p* approaches 1, triggering SMA’s global exploration mode. Meanwhile, the weight coefficient *W* is calculated using Equation 9, with a value range of 1–2. A larger *W* value makes slime mould individuals more inclined to move towards randomly selected positions, quickly traversing the entire VMD hyperparameter space and effectively adapting to the wide-range characteristics of the DTR data hyperparameter space. In the late stages of iteration, as optimization progresses, the population fitness gradually converges (the gap between *S*(*i*) and *DF* narrows), and the value of *p* approaches 0, causing the algorithm to automatically switch to the local exploitation mode. At this point, the *W* value is dynamically adjusted through the difference between the current optimal and worst fitness values in Equation 9, converging to around 1. Slime mould individuals are more inclined to approach the optimal position *X_b_*(*t*), focusing on the local optimal region of the hyperparameter space. This switch is fully driven by the envelope entropy of decomposed DTR data (i.e., the fitness function) without manual intervention, perfectly matching the dynamic changes of DTR data from "severe fluctuations" to "relative stability", and ensuring that the optimal solution can still be quickly locked in during the migration of the hyperparameter space.

**1.2 Avoiding Local Optimal Solutions**

(1) Particle Swarm Optimization (PSO) Algorithm.

The particle position update of PSO relies on the dual guidance of "personal best solution (*pbest*) + global best solution (*gbest*)", and its update formula is as follows:

$\begin{matrix} X_{i}(t+1)=X_{i}(t)+c_{1}r_{1}(pbest_{i}-X_{i}(t))+ \\ c_{2}r_{2}(gbest-X_{i}(t)) \end{matrix}$(1)

Among them, the learning factors *c_1_* and *c_2_* are fixed values, and the search direction of particles is restricted within the joint region of their own historical optimal and the global optimal. This mechanism is efficient in static unimodal hyperparameter spaces but exhibits significant flaws in the dynamic multimodal space of Dynamic Thermal Rating (DTR) data: When DTR data switches from high-frequency fluctuations to long-term stability, the global optimal solution of the hyperparameter space migrates from a narrow-bandwidth, high-penalty region to a wide-bandwidth, low-penalty region. At this point, the *gbest* (global best) of PSO (Particle Swarm Optimization) has been locked by the local optimal solution of the previous stage. Due to the strong guidance of *gbest*, particles are difficult to deviate from the original search direction to explore new optimal regions; in addition, the population diversity of PSO is only maintained by random numbers *r_1_* and *r_2_*, lacking an active diversity regulation mechanism. When there are multiple suboptimal hyperparameter combinations in DTR data (such as local optima corresponding to different wind speed intervals), particles tend to quickly aggregate around a certain suboptimal solution, resulting in premature convergence of the population and failure to jump out of the local optimal trap.

(2) Grey Wolf Optimizer (GWO) Algorithm

GWO achieves optimization by simulating the encircling-hunting behavior of wolf packs. Its position update relies on the guidance of α (global optimal), β (suboptimal), and δ (third optimal), with the formula as follows:

$\begin{matrix} X(t+1)=X_{\alpha}(t)-A_{1}\cdot|C_{1}\cdot X_{\alpha}(t)-X(t)|+ \\ X_{\beta}(t)-A_{2}\cdot|C_{2}\cdot X_{\beta}(t)-X(t)|+X_{\delta}(t)- \\ A_{3}\cdot|C_{3}\cdot X_{\delta}(t)-X(t)| \end{matrix}$(2)

Among them, the contraction factor A linearly decreases with iterations, resulting in the following flaws in the DTR data scenario:

First, the population diversity decays too rapidly: the hierarchical order of α, βand δ is fixed, and other individuals only act as followers, lacking independent exploration capabilities. When there are multiple local optima in the hyperparameter space, the wolf pack is easily attracted to the local optimal region where α resides, rapidly losing population diversity and failing to explore other potential optimal solutions.

Second, the rigid contraction of the search range: the linear decay mechanism of the contraction factor A is irrelevant to the dynamic changes of the DTR data hyperparameter space. For instance, when DTR data suddenly exhibits high-frequency fluctuations in the mid-iteration stage and the hyperparameter space expands abruptly, A of GWO has already decreased to a small value, and the search range has contracted. As a result, it is difficult to escape the original local optimal region to adapt to the new hyperparameter space.

(3) Slime Mould Algorithm (SMA)

Based on the "foraging-encircling-oscillating" behavior of slime mould, SMA employs a triple mechanism to avoid local optima in the dynamic multimodal hyperparameter space of DTR data and achieve deep adaptation to data characteristics:

Dynamic balance between random exploration and directed exploitation: Driven by the control parameter p, in the early stage of iterations when p approaches 1, the algorithm performs global exploration. Slime mould individuals randomly select two individuals *X_A_*(*t*) and *X_B_*(*t*) for position update via the following formula, without relying on historical optimal solutions. This effectively maintains population diversity and covers multiple potential optimal regions in the DTR data hyperparameter space.

$X(t+1)=X_{b}(t)+v_{b}\cdot(W\cdot X_{A}(t)-X_{B}(t))$(3)

In the late stage of iterations, when *p* approaches 0, the algorithm switches to local exploitation. However, it still retains a certain exploration capability through the random disturbance term $v_{c}\cdot X(t)(r\geq p)$, thus avoiding falling into local optima;

Diversity Regulation via the Adaptive Weight *W*: *W* is dynamically adjusted based on the difference between the optimal and worst fitness values ($bF-wF$) of the current population. When the population approaches a local optimal solution (i.e., when $bF-wF$ decreases), *W* converges to around 1. Meanwhile, *v_b_* decays with iterations but retains random oscillation characteristics, simulating small fluctuations in the cytoplasmic flow of slime mould—this drives individuals to slightly deviate from the current region and explore adjacent potential optimal solutions. When the hyperparameter space migrates (e.g., when DTR data switches from stability to fluctuation), $bF-wF$ increases, and the value of *W* expands to the range of 1~2, reactivating global exploration and avoiding constraint by old local optimal solutions.

Mutation Mechanism of Biological Oscillators: SMA simulates the biological oscillators of slime mould via the random oscillations of *v_b_* and *v_c_*, whose value ranges are dynamically adjusted with iterations. Even in the mid-iteration stage, small mutations can still be generated to help the population escape the discovered local optimal solutions. This mutation is indirectly driven by the fitness function (envelope entropy of DTR components). When the decomposition performance is poor (i.e., the fitness value is high), the value of a maintains a large range, increasing the mutation probability and ensuring the algorithm can still explore new optimal regions during the dynamic migration of the hyperparameter space.

**2. Experimental Aspect**

Section 4.3 of the original paper has constructed a complete comparative experiment system targeting two core indicators: convergence speed and optimization accuracy. The experiment uniformly sets the population size to 20, the maximum number of iterations to 20, and the optimization ranges as K∈[3,12] andα∈[200,2600]; the fitness function is the minimum envelope entropy of DTR components. The comparative algorithms cover two categories (a total of 5 classic algorithms): non-population-based (Simulated Annealing (SA), Tabu Search (TS)) and population-based (PSO, WOA, GWO).

The experimental results show that in terms of convergence speed: SMA drops rapidly from the initial fitness value of 8.52 and stabilizes at 8.39 after only a few iterations, improving the convergence efficiency by 30% to 50% compared to PSO (initial value 8.89, slow decline), GWO (slow change in the early stage), and WOA (significant drop only at the 5th iteration). In terms of optimization accuracy: the stabilized fitness value of SMA (8.39) is significantly lower than that of PSO (8.79), GWO (8.49), WOA (8.48), SA (8.86) and TS (8.80), proving that it can find better VMD hyperparameter combinations. The above experiments have initially verified the advantages of SMA in optimization performance.

To further rule out the randomness of algorithm selection, we added a swarm intelligence algorithm widely used in the field of parameter optimization in recent years—the Atlantic Puffin Optimization (APO)—as a supplementary comparative object. The experimental settings are exactly the same as those in Section 4.3 of the original paper (population size, number of iterations, optimization range, and fitness function remain unchanged).

The APO algorithm achieves optimization by simulating the foraging and breeding behaviors of Atlantic puffins, with core mechanisms including group scattered exploration, individual precise dive predation, and population update and iteration. However, the optimization logic of this algorithm relies on fixed behavioral pattern switching, lacking an adjustment mechanism that dynamically adapts to the characteristics of DTR data. The supplementary experimental results show that: in terms of convergence speed, SMA stabilizes after only 5 iterations, while APO requires 8 iterations to stabilize (as it needs to go through the fixed process of "group scattered exploration-individual positioning-dive predation"), which is 37.5% slower than SMA; even WOA, the best-performing comparative algorithm in the original paper, requires 10 iterations to stabilize, and SMA is 50% faster than it.

In terms of optimization accuracy, the final fitness value of SMA in Dataset 1 is 8.39, which is lower than APO’s 8.41. This difference stems from the fact that APO’s "dive predation" mechanism tends to quickly lock onto the current optimal region, but struggles to adapt to the dynamic migration of the DTR hyperparameter space—resulting in the optimization result falling into local suboptimality rather than global optimality.

Combining the existing experiments in the original paper and the supplementary verification of the newly added APO algorithm, it can be seen that: compared with algorithms such as PSO, GWO, and APO, SMA has faster convergence speed and higher optimization accuracy in the DTR data scenario, and can effectively avoid local optima. Its dynamic exploration-exploitation mechanism is highly adapted to the non-stationary characteristics of DTR data and the dynamic changes of the hyperparameter space.

**Question 2:**

This study constructed an integration of the Elman and TCN models. What is the specific theoretical basis for choosing these two specific models for integration? Before finalizing this architecture, were other potential candidate models (such as LSTM, GRU) considered and benchmarked?

**Reply to Question 2:**

Thank you very much for your valuable comments on this study. Your point about the theoretical basis for the integration of Elman and TCN, as well as the consideration of candidate models and benchmark validation, precisely hits the core logic of the model architecture design. Based on your suggestions, we have systematically sorted out the theoretical support for model selection and the limitations of candidate models starting from the essential characteristics of DTR data, and supplemented relevant literature evidence. The specific thoughts and replies are as follows:

The fundamental logic of model integration is the adaptability between data characteristics and model architecture. Only by first clarifying the essential characteristics of DTR data can we design a targeted integrated architecture that covers these characteristics. According to the thermal balance principle of transmission lines, DTR data is essentially a multi-scale temporal feature driven by the coupling of multiple meteorological factors, which specifically exhibits two core characteristics: 1、 Short-term high-frequency volatility: DTR is affected by micro-meteorological factors such as sudden wind speed changes and short-term temperature fluctuations, showing sharp mutations over short time scales. Previous studies have pointed out that such short-term mutations are the key driver of line overheating risks, requiring the model to have the ability to capture features on short time scales. 2、 Long-term multi-scale periodicity: DTR is dominated by solar radiation (annual cycle) and ambient temperature (seasonal cycle), showing periodic trends over long time scales (e.g., years, seasons). Existing studies have shown that long-term periodic features are the core basis for dynamic capacity increase to ensure maximum transmission capacity, requiring the model to have the ability to extract features on long time scales. Therefore, the selection of a single model in the integrated framework needs to simultaneously resolve the contradiction between short-term mutation capture and long-term periodic extraction, while avoiding biased omission of certain features by a single model.

The integration of Elman and TCN is precisely based on the accurate matching of the above DTR data characteristics. Their inherent architectural advantages form a natural complement, and relevant studies have verified their effectiveness in temporal prediction.

**(1) Selection basis for the Elman model**

The Elman model adopts a closed-loop recursive structure consisting of the input layer, hidden layer, and context layer. It directly feeds back the hidden layer state at the previous time step to the current input, making the model highly sensitive to feature correlations between adjacent time steps. This exactly matches the temporal continuity feature of DTR’s short-term high-frequency fluctuations.

The uniqueness of the Elman model lies in introducing the context layer to construct a local closed loop of input-hidden-context, and its mathematical expression can be decomposed as:

Hidden layer state update: $h_{t}=\sigma(W_{ih}x_{t}+W_{hh}h_{t-1}+b_{h})$

Context layer state feedback: $c_{t}=\gamma\cdot h_{t-1}+(1-\gamma)\cdot c_{t-1}$

Output layer calculation: $y_{t}=\sigma(W_{ho}h_{t}+b_{o})$

The roles of the key parameters and mechanisms are as follows:

1、Context layer feedback coefficient $\gamma$: It is usually set to 0.8~0.95, meaning that the context layer retains 80%~95% of the hidden layer stat $h_{t-1}$ at the previous time step, and only slightly integrates the context information $c_{t-1}$ from earlier time steps. This short memory window design enables the model to have almost undiminished sensitivity to feature correlations between adjacent time steps (e.g., DTR data at time t and t-1. Meanwhile, the short-term high-frequency fluctuations of DTR data are exactly characterized by strong correlations between adjacent sampling points and a sharp drop in correlation after one sampling point interval—this forms an accurate mathematical-level matching between the two.

2、Gating-free direct feedback mechanism: Unlike the forget gate-input gate structure of LSTM and GRU models, the context layer of the Elman model does not need to filter historical states through the sigmoid activation function; the hidden layer state $h_{t-1}$ at the previous time step directly participates in the calculation of the current hidden layer $h_{t}$. This delay-free feedback significantly improves the model’s response speed to short-term mutations in DTR data. For example, when the wind speed surges from 1m/s to 5m/s, the Elman model can capture the upward trend of DTR data within 1 sampling step, while the LSTM model requires 2~3 sampling steps to fully respond due to gating-based filtering.

In fact, in other prediction domains with data characteristics similar to those of DTR, researchers have long proposed using the Elman model to extract short-term high-frequency fluctuation features of data for more accurate prediction results. In their paper 《Prediction of New Energy Power Generation and Load Demand in New Power Systems Based on the EMD-GM-Elman Neural Network Combination Model》^[1]^, Zhao Hanchao et al. adopted the Elman model to capture power correlations at 15-minute intervals, targeting the short-term high-frequency fluctuations in new energy power generation data. The results show that the Elman model outperforms both the traditional grey model and the CNN-LSTM combination model in predicting new energy power generation. The core reason is that the context layer of the Elman model can retain the power mutation features of the previous moment without delay, whereas the gating mechanism of LSTM filters out part of such mutation information.

To summarize, the Elman model is highly suitable for DTR data prediction, primarily due to the deep matching between its characteristics and the short-term high-frequency fluctuation features of DTR:

1、The short memory window design accurately captures DTR’s features (strong correlations between adjacent sampling points and correlation attenuation within short cycles), avoiding interference from invalid historical information;

2、The gating-free direct feedback mechanism can quickly respond to DTR’s abrupt changes caused by gusts or short-term strong radiation, reducing the response delay by 2–3 sampling steps compared to LSTM and GRU.

Moreover, this adaptability has been verified by previous researchers in prediction domains involving similar high-frequency mutation data (e.g., new energy power). Thus, the Elman model becomes the optimal choice for the short-term feature extraction module in the integrated DTR prediction architecture.

**(2) Selection Basis for the TCN Model**

The TCN model constructs a feature extraction network capable of covering multi-scale temporal information through a combined architecture of dilated causal convolution and residual connections. This architecture correlates and fuses historical states across different time scales, endowing the model with an efficient ability to capture temporal trends over long cycles—this exactly matches the characteristic of long-term multi-scale periodicity of DTR data. The core feature of the TCN model lies in using dilated causal convolution to expand the receptive field, while alleviating the vanishing gradient problem of deep networks via residual blocks. The mathematical expressions of its key structures can be decomposed as follows:

Dilated Causal Convolution Calculation: $x_{t}^{(l)}=\sigma\left(\sum_{k=0}^{K-1} W_{k}^{(l)}\cdot x_{t-d^{(l)}\cdot k}^{(l-1)}+b^{(l)}\right)$

Residual Block Output: $y_{t}^{(l)}=x_{t}^{(l)}+x_{t}^{(l-1)}$

Output Layer Calculation: $y_{t}=\text{Linear}\left(x_{t}^{(L)}\right)$

Here, l denotes the number of network layers, $d^{(l)}=2^{l-1}$ is the dilation factor of the l-th layer, K is the convolution kernel size, $W_{k}^{(l)}$ is the k-th weight of the convolution kernel in the l-th layer, and $b^{(l)}$ is the bias.

The roles of the key parameters and mechanisms are as follows:

1、Dilation factor and receptive field expansion: The dilation factor of TCN grows exponentially with the number of layers, enabling the receptive field to expand rapidly in the form of $K+(K-1)(d^{(1)}+d^{(2)}+\cdots+d^{(L)})$. For example, when the convolution kernel $K=3$ and the number of layers $L=3$, the receptive field can reach 17 sampling steps; when $L=6$, the receptive field covers 97 sampling steps, which can fully capture the periodic features of DTR. This design exactly matches DTR’s characteristic of overlapping multi-scale long-term trends (e.g., daily and seasonal cycles). Unlike LSTM and GRU models that rely on multi-layer stacking (LSTM requires stacking over 8 layers to achieve the same receptive field), this avoids the vanishing gradient problem.

2、Causal convolution and temporal no-leakage: The causal convolution structure of TCN restricts the convolution operation to only use current and previous historical data, ensuring that feature extraction at time *t* depends solely on information prior to time t. This fully meets the temporal no-leakage engineering requirement for DTR prediction. While LSTM also satisfies causality, the historical information filtering of its gating mechanism tends to lose weak correlation features over long cycles.

In fact, in other prediction domains with data characteristics similar to those of DTR, researchers have long proposed using the TCN model to extract the long-term multi-scale periodic features of data for more accurate prediction results. In their paper 《Ultra-Short-Term Wind Power Prediction Based on Information Reorganization and TCN-LSTM-MHSA》^[2]^, researchers such as Chen Lei adopted the TCN model to extract long-term features, targeting the composite characteristics of long-period fluctuations and multi-day trends in wind power. The results show that the TCN model achieves higher accuracy than LSTM in predicting the multi-scale long-term trends of wind power. The core reason is that the dilated convolution of TCN can cover trends across different time scales simultaneously, while the long-memory dependence of LSTM is prone to interference from short-term fluctuations.

To summarize, the TCN model is highly suitable for DTR data prediction, primarily due to the deep matching between its characteristics and the long-term multi-scale periodic features of DTR:

1、The exponential expansion of the receptive field via dilated convolution accurately covers DTR’s multi-scale long-term trends (such as daily and seasonal trends), avoiding the gradient issues caused by multi-layer stacking in LSTM and GRU;

2、The causal convolution structure ensures temporal no-leakage, which aligns with the engineering reliability requirements for DTR prediction.

In addition, This adaptability has been verified in similar multi-scale temporal prediction domains (e.g., wind power prediction), thus making it the optimal choice for the long-term feature extraction module in the integrated DTR prediction architecture.

**(3) Reasons for Not Selecting LSTM and GRU Models as Benchmark Models**

Although LSTM, GRU, and TCN are all used for long temporal modeling, the architectural design of LSTM and GRU is inherently less suitable than TCN for adapting to the long-term multi-scale periodic superposition features of DTR data. The specific comparisons are as follows:

1、Limitations of the Receptive Field Expansion Mechanism

LSTM and GRU models need to expand the receptive field by stacking network layers (e.g., stacking more than 8 layers to cover the 24-hour daily cycle of DTR). However, increasing the number of layers causes vanishing gradients (the partial derivative $\frac{\partial L}{\partial W}$ of the loss function with respect to deep-layer weights decays exponentially), making it impossible to capture multi-scale features (such as the 24-hour daily cycle and 90-day seasonal cycle) simultaneously. This conflicts with the core requirement of DTR data’s multi-scale long-period features. In contrast, the TCN model achieves exponential expansion of the receptive field via dilated causal convolution: it can cover 29 sampling steps with only 3 layers and 97 sampling steps with 6 layers. It does not rely on multi-layer stacking, has more stable gradients, and can fully cover the multi-scale cycles of DTR.

2、Disadvantages in Computational Efficiency

LSTM and GRU models have a large number of parameters due to the high number of stacked layers, leading to longer training time—this fails to meet the engineering requirement of near-real-time scheduling for grid DTR prediction. In contrast, the lightweight dilated convolution architecture of the TCN model reduces the parameter count, and its training time is significantly shorter than that of LSTM with the same receptive field, making it better suited to the efficiency requirements of DTR prediction.

All the above analyses are qualitative. To more effectively demonstrate that the TCN model is more suitable for DTR data prediction than the LSTM and GRU models, we have already conducted a comparative experiment between TCN and GRU in the original paper, verifying the advantages of TCN—details can be found in Figure 6 of Section 4.4 “Comparative Experiments” in the original paper. However, considering that the GRU model is a lightweight variant of the LSTM model, there might be an unexpected scenario where the LSTM model achieves higher prediction accuracy. To avoid this scenario, we have supplemented a comparative experiment between TCN and LSTM in Section 4.4 “Comparative Experiments” of the original paper; the detailed content can be found in the highlighted text in Section 4.4 of the paper.

In summary, both in terms of the adaptability of architectural principles and the results of quantitative experiments, LSTM and GRU are inferior to TCN in extracting the long-term multi-scale periodic features of DTR. Thus, we finally selected the TCN model as the long-term feature module of the integrated architecture. Thank you again for your professional suggestions—this adjustment has significantly improved the targeting and rigor of the model selection demonstration!

**References**

[1] Zhao HCH, Cong ML, Liu J, Han ZY, Hu NN, Pan GY, et.al. Forecasting of the New Energy Generation and Load Deman in the Novel Power System Based on EMD-GM-Elman Neural Network Combined Model [J]. Power System and Clean Energy, 2024, 40(10): 132-141.(in Chinese)

[2] Chen K, Huang KY, Zhang Y, Cai KZ, Chen Y, Zhang ZR. Ultra-short-term wind power forecasting based on information reorganization and TCN-LSTM-MHSA. Southern Power System Technology, 1-10[2025-10-15]. https://link.cnki.net/urlid/44.1643.TK.20240626.1553.009. (in Chinese)

**Question 3:**

The dynamic weighting mechanism based on grey correlation degree is a key contribution. Could the author elaborate on the computational overhead introduced by this point-by-point weighting calculation, especially regarding its feasibility for real-time or near real-time DTR prediction in practical power system operations?

**Reply to Question 3:**

Thank you sincerely for your valuable comments! The "computational overhead of the dynamic weighting mechanism based on grey relational grade" and the "feasibility of real-time/near-real-time DTR prediction in actual power systems" that you are concerned about are the core of measuring the engineering value of this innovation point. Combining the mechanism principle, experimental testing, and power system engineering practice, we provide the following detailed explanation:

First, it is necessary to clarify the phase division between the offline training and online real-time prediction of the model in this paper—this is also the core premise to ensure current real-time performance. The large-scale data training of the model (e.g., optimizing SMA-VMD hyperparameters and training the Elman and TCN networks using months of historical data) belongs to the offline preparation phase. This phase takes about 1 hour, and applies only to the two datasets of the model in this paper. Moreover, it only needs to be completed once before model deployment, or updated slightly quarterly/annually to adapt to seasonal characteristic changes; its time consumption is completely excluded from online real-time prediction.

For DTR real-time prediction in actual power systems, a lightweight sliding window strategy is adopted. The window only contains recent historical data (e.g., this paper adapts to a 15-minute sampling interval, and the window length is set to 20 time steps, corresponding to 300 minutes of recent data). During each prediction, there is no need to recall all training data; only the pre-trained offline model parameters are loaded. The grey relational coefficient and dynamic weights are calculated based on the recent predicted values and true values within the window, and then the prediction result is output by combining real-time monitored micro-meteorological data. The window slides automatically with the prediction cycle: the oldest set of data is removed and the latest set is added. This not only ensures the adaptability of weights to the latest DTR changes, but also controls the online computation data volume to a very small range. Actual tests show that the single-time consumption of the current online prediction process is stably around 750 seconds, which fully meets the engineering requirements of 15-minute rolling prediction and verifies the near-real-time feasibility of this mechanism.

Second, we have also taken into account that data intervals will become more granular in the future, which is indeed a challenge that requires forward-looking responses in the DTR prediction field. With the development of micro-meteorological monitoring technology, future data sampling intervals may be shortened from 15 minutes to 5 minutes or even 1 minute. The surge in data volume will place higher demands on the real-time performance of online prediction. To address this potential issue, we have initially planned two optimization directions to ensure the adaptability of the method under shorter prediction intervals:

1、Model lightweight pruning strategy: The current Elman and TCN networks contain some redundant parameters, such as the edge convolution kernels of TCN and the neurons in the Elman hidden layer that contribute minimally to short-term features. In the future, structured pruning technology can be adopted: via L1 regularization, redundant parameters (those that affect prediction accuracy by ≤1%) will be identified and removed. It is estimated that this can reduce the model parameter count by 30%, further lowering the computational complexity of online inference to adapt to the prediction requirements of shorter minute intervals in the future.

2、Parallel scheduling of computing tasks: In the future, the three modules (VMD decomposition, dual-model prediction, and dynamic weighting) can be decomposed into independent parallel tasks and deployed on the multi-core hardware of edge computing nodes. For example, while the Elman model processes short-term features, the TCN simultaneously extracts long-term features; VMD decomposition and dynamic weighting also occupy independent cores respectively. Task scheduling avoids idle computing resources and reduces data transmission latency between different modules, making it particularly suitable for high-frequency data input scenarios.

In summary, the method in this paper currently meets the near-real-time requirements of 15-minute rolling prediction through the “offline training + online sliding window” mechanism. In response to the future challenge of more granular data intervals, we have also planned feasible optimization paths—this not only ensures current engineering feasibility but also reserves room for subsequent research. We kindly request you to review this again; if you have any other suggestions, we will continue to refine the work.

**Question 4:**

The manuscript indicates that compared with the single TCN model and the single Elman model, the integrated model has improved the accuracy by 22.91% and 27.95% respectively. The author needs to make a comparison with the high-level research work published recently.

**Reply to Question 4:**

Thank you sincerely for your valuable comments! Your suggestion that “this study should further compare with high-level research findings published in recent years” is of great significance for enhancing the novelty and academic positioning of our research. We attach great importance to this and have carefully reviewed existing studies; here is our detailed response:

First, we would like to clarify that in Section 4.6 “Model Generalization Test Experiment” of the original paper, we have already conducted a systematic comparison with high-level studies in the DTR prediction field in recent years. The selected comparison objects are:

Reference [5], published in ***Proceedings of the CSU-EPSA***, using the method of EMD + Bayesian optimization + BiLSTM;

Reference [15], published in ***Proceedings of the CSU-EPSA***, using the method of AL-BILSTMDN;

Reference [28], published in ***Shandong Electric Power***, using the method of EMD + DBO + BiLSTM.

These references are cutting-edge achievements published between 2022 and 2024, and their core methods cover decomposition algorithms, deep learning, hyperparameter optimization, etc.—to some extent, they represent the mainstream technical paths of current DTR prediction. The comparison results have fully demonstrated the performance advantages of our model.

To further respond to your suggestion and enhance the comprehensiveness of the comparison, we have added another recent high-level study: Reference [18], published in ***Proceedings of the CSU-EPSA***, using the Transformer + CNN + AR model. Its technical route is directly comparable to our research. The new comparison strictly follows the three-identical principles (identical data, identical evaluation metrics, and identical experimental environment) to ensure the fairness of the comparison.

Meanwhile, we have added the following analysis content to the existing analysis section in Section 4.6 of the original paper: “The model in Reference [18] adopts an embedded combination of Transformer, CNN, and AR modules to handle the nonlinear and linear fitting relationships of DTR data. However, in Dataset 1, the short-term mutations of DTR data and the strong non-stationarity of meteorological parameters impose stricter requirements on the model’s ability to capture dynamic features. The DT-AR model in Reference [18] relies on the Transformer attention mechanism and the AR module: the former tends to blur the time position sensitivity when processing temporal data, while the latter struggles to fit nonlinear mutation relationships. Moreover, no targeted non-stationary data processing module is designed, resulting in an RMSE of 19.9743.” The addition of this analysis content is mainly to elaborate on the performance differences between our model and the model in Reference [18].

Thank you again for your rigorous review and valuable guidance. The revised comparative experiments are more comprehensive and in-depth, fully demonstrating the academic value and technical advancement of this study. We kindly request your further review!

**Question 5:**

Regarding the generalization ability, this method was tested on "different datasets". The author needs to provide more details about these datasets in order to better evaluate the claimed generalization ability.

**Reply to Question 5:**

Thank you sincerely for your valuable comments! Your suggestion to "supplement detailed dataset information to better evaluate generalization ability" is highly targeted. We attach great importance to this and have carried out refined supplementation to the relevant sections of the original paper. Here is our response regarding the specific revision details:

The generalization experiments corresponding to different datasets (which you are concerned about) have been conducted in Section 4.6 "Model Generalization Test Experiment" of the original paper. This revision focuses on supplementing key details of the two datasets, including environmental differences, meteorological characteristics, data scale, and distribution properties. This allows the differences between the datasets to be quantified and visualized, making it easier to evaluate the model’s adaptability in complex scenarios.

Specifically, the revision is implemented at the beginning of Section 4.6 of the original paper, where a ~200-word description of dataset details (highlighted in the original paper) is added. The supplementary content includes: the specific sample sizes of the two datasets, topographical differences along the transmission lines they cover, statistical characteristics of core meteorological parameters (temperature, wind speed, solar radiation), and the dominant variation patterns of DTR data. It also clearly explains why these differences can effectively test the model’s generalization ability—by simulating two typical scenarios ("high-temperature and strong fluctuations in summer" and "low-temperature and stable changes in late winter and early spring"), and covering the differences between the urban heat island effect and suburban natural meteorological environments, the comprehensiveness and persuasiveness of the generalization test are ensured.

Additionally, we have retained the original comparative experiment design and result analysis in this section. The supplemented dataset details form a logical closed loop with the subsequent experimental results, further confirming the generalization advantage of our model: it can maintain a prediction accuracy of over 95% under different meteorological conditions and topographical environments. This revision does not change the experimental conclusions of the original paper; it only enhances the transparency and rigor of the study by supplementing key details. We kindly request your further review!

**Question 6:**

The proposed method involves multiple complex stages (SMA-VMD decomposition, dual-model training, dynamic weighting). The author needed to conduct an ablation study to quantify the individual contribution of each component (for example, the improved VMD versus the standard VMD, the integrated model versus the single best model) to the overall performance gain.

**Reply to Question 6:**

Thank you sincerely for your valuable comments on this paper. Your suggestion that "ablation experiments should be conducted to quantify the individual contributions of components (such as SMA-VMD decomposition, dual-model training, and dynamic weighting) to the overall performance" aligns perfectly with the core direction of our initial experimental design. Before you put forward this suggestion, we had already conducted basic comparative experiments focusing on "improved VMD vs. standard VMD" and "integrated model vs. optimal single model". However, your guidance made us realize that we need to further refine variable control and enhance the quantification accuracy of contributions. Therefore, we have added three more focused verification experiments based on the original experiments—this not only continues the previous verification logic but also more systematically addresses your rigorous requirement for "individual contributions of each component". We now report the specific details as follows:

In Section 4.5 "Ablation Experiments" (corresponding to Figures 9 and 10), we have verified the difference between "improved VMD and standard VMD": the improved VMD reduces the sample entropy of the subsequences after DTR data decomposition, and the R² of the corresponding integrated model increases from 89.64% to 95.91%. In addition, in Section 4.4 "Comparative Experiments" of the paper, we have also verified the comparison between our model and the optimal single model (Transformer model): the R² of our integrated model reaches 95.91%, which is a 21.02% increase compared to the R² of the optimal single model (Transformer model). The relevant results have been presented in the performance metrics of Figures 7 and 8.

However, your suggestion made us realize that we need to further refine variable control and enhance the independent quantification accuracy of each component’s contribution. Therefore, we have added three focused verification experiments based on the original experiments, supplemented them to the corresponding sections, and updated the figures and tables:

First, we added a comparison between our model and the single LSTM model in Section 4.4’s comparative experiments, to quantify the contribution of the integrated model relative to the optimal single model: the experiment controls for identical datasets, input features, and training parameters, with only the model architecture as the variable (our model is the SMA-VMD + Elman-TCN time-varying integrated model, while the comparison model is a single LSTM). The results show that, as a classic model, LSTM has strong ability to capture long-term temporal dependencies via gating structures like forget gates and input gates, but it also lacks optimization modules designed for the non-stationary characteristics of DTR data—thus its prediction performance is still far inferior to our model: LSTM’s R² is only 67.91%, 28.0% lower than that of our model; its RMSE is as high as 59.1816, 69.2% lower than that of our model. This result further confirms that relying solely on temporal modeling capability is insufficient to adapt to the non-stationary fluctuation characteristics of DTR, and a targeted mechanism to mitigate non-stationary features must be introduced. It intuitively verifies that our model achieves a significant performance breakthrough compared to single models, through the synergistic capture of short-term fluctuations and long-term trends.

Second, we added a comparison between our model and the VMD-non-time-varying integrated model in Section 4.5’s ablation experiments, to verify the individual contribution of the dynamic weighting mechanism. The results show that the R² of our model is 3.49% higher than that of the comparison model, quantifying the independent value of this core component.

Finally, we added a comparison between our model and the method in Reference [18] in the generalization test of Section 4.6, to further verify the advantages of our model: Reference [18] adopts an embedded combination of the Transformer model, CNN algorithm, and AR model. The results show that the R² of our model is 4.72% higher than that of Reference [18] in Dataset 1, and 5.73% higher in Dataset 2—this strengthens the improvement significance of each component from the perspective of external method reference.

Through the above-added experiments, we have systematically quantified the individual contributions of each component, making the verification logic of the study more rigorous. We have updated the experimental section of the full paper in accordance with the journal format. We kindly request your re-review, and we will continue to refine the work if you have any other suggestions.

**# Reviewer 2**

**Question 1:**

It is recommended to supplement the comparison with other emerging forecasting technologies to more comprehensively demonstrate its innovation and advantages.

**Reply to Question 1:**

Dear Reviewer, Thank you very much for your valuable suggestion to "supplement comparisons with other emerging prediction technologies." This suggestion provides a key direction for further verifying the innovativeness and advantages of our research method. We have incorporated your opinion and added comparative experiments with emerging technologies based on the existing comparisons in the original paper. Here is our response regarding the specific details:

In the initial research design, we were already aware of the necessity of this issue. Therefore, in Section 4.6 of the original paper, we selected methods from 3 representative papers published in 2021–2025 in core journals in the power field (e.g., 《***Proceedings of the CSU-EPSA***》 and 《***Shandong Electric Power***》) as the comparison benchmarks. Specifically, these include:

Reference [5], published in ***Proceedings of the CSU-EPSA***, using the method of EMD + Bayesian optimization + BiLSTM;

Reference [15], published in ***Proceedings of the CSU-EPSA***, using the method of AL-BILSTMDN;

Reference [28], published in ***Shandong Electric Power***, using the method of EMD + DBO + BiLSTM.

These references are cutting-edge achievements published in 2021–2025, and their core methods cover decomposition algorithms, deep learning, hyperparameter optimization, etc.—to a certain extent, they represent the mainstream technical paths of current DTR prediction. The comparison results have fully demonstrated the performance advantages of our model.

In addition, to further respond to your suggestion and enhance the comprehensiveness of the comparison, we have added another recent high-level study: Reference [18], published in 《***Proceedings of the CSU-EPSA***》, using the Transformer + CNN + AR model. Its technical route is directly comparable to our research. The new comparison strictly follows the three-identical principles (identical data, identical evaluation metrics, identical experimental environment) to ensure the fairness of the comparison.

Meanwhile, we have added the following analysis content after the existing analysis section in Section 4.6 of the original paper: "The model in Reference [18] adopts an embedded combination of Transformer, CNN, and AR modules to handle the nonlinear and linear fitting relationships of DTR data. However, in Dataset 1, the short-term mutations of DTR data and the strong non-stationarity of meteorological parameters impose stricter requirements on the model’s ability to capture dynamic features. The DT-AR model in Reference [18] relies on the Transformer attention mechanism and the AR module: the former tends to blur the time position sensitivity when processing temporal data, while the latter struggles to fit nonlinear mutation relationships. Moreover, no targeted non-stationary data processing module is designed, resulting in an RMSE of 19.9743." The addition of this analysis content is mainly to elaborate on the performance differences between our model and the model in Reference [18], which can be found in the highlighted text in Section 4.6 of the paper.

In summary, through the verification of our model against cutting-edge technical methods, the innovativeness and engineering advantages of our research method have been more comprehensively supported. Thank you again for your professional guidance—this supplementation has not only improved the demonstration framework but also highlighted the technical value of our method in the field of DTR prediction. We have revised the original paper in accordance with the above content and look forward to your further review.

**Question 2:**

In the method description section, it is suggested to elaborate on the specific implementation details of the slime mold algorithm in optimizing VMD hyperparameters, including the convergence analysis of the algorithm and the rationality of parameter selection.

**Reply to Question 2:**

Dear Reviewer, Thank you sincerely for your valuable suggestions on this paper. Your proposal to "refine the specific implementation details of the Slime Mould Algorithm (SMA) for optimizing VMD hyperparameters, algorithm convergence analysis, and rationality of parameter selection" precisely targets the reproducibility of the research method, helping us address the shortcoming of incomplete process description in the original method. Following your guidance, we have supplemented the full-process details, convergence data, and parameter verification experiments in the corresponding sections of the original paper. We now report the specific revision content as follows:

**(1) Supplementing specific implementation details of SMA optimizing VMD hyperparameters**

To clarify the reproducible steps of SMA optimizing VMD hyperparameters, we added "2.2.3 Specific Process of SMA Optimizing VMD Hyperparameters" in Section 2.2, which breaks down the core steps from hyperparameter space definition to iteration termination in detail:

1. Hyperparameter search space definition**: Clarify the ranges of the two hyperparameters (number of decomposition components and penalty factor), and determine the approximate boundaries of the hyperparameters based on DTR data characteristics and pre-experiments to avoid invalid hyperparameters;

2. Fitness function calculation**: Based on information entropy theory, take the envelope entropy of components after VMD decomposition as the optimization objective;

3. Generation of SMA initial population**: Based on swarm optimization theory, generate an initial SMA population that covers the hyperparameter space to ensure the global search capability of the SMA algorithm;

4. Iterative update of SMA individual positions**: Follow the biological mechanism of SMA ("slime mould foraging-encircling-grabbing"), update the population positions through mathematical models, and balance global exploration and local exploitation;

5. Physical constraint verification and fitness recalculation of updated parameters**: Correct the parameters that violate VMD physical constraints during iterative updates to ensure parameter validity and avoid invalid computation;

6. Convergence criterion and optimal hyperparameter output**: Based on numerical optimization theory, determine the optimization termination status according to the set convergence criterion, balancing optimization accuracy and engineering real-time performance;

**(2) Rationality of Parameter Selection**

When the SMA algorithm is used for VMD hyperparameter optimization, it has three main core parameters: population size and maximum number of iterations. The selection of these core parameters is not a subjective setting, but a rational choice based on DTR data characteristics and optimization accuracy.

1、Population Size

Population size is a key parameter that balances optimization accuracy and computational efficiency. A too-small population size will lead to insufficient coverage of the search space, which easily causes the SMA algorithm to fall into local optimum; a too-large population size will increase the iterative computation load, violating the near-real-time engineering requirements of DTR data prediction. This parameter setting is mainly verified step by step through multiple rounds of parameter sensitivity tests.

Because the population size is an integer-type parameter, the research method in this paper mainly obtains the optimal parameter through multiple groups of parameter sensitivity tests. This ensures that the finally determined population size can both enable individuals to cover the key regions of the hyperparameter space and control the time consumption of a single optimization within an engineering-acceptable range. Specifically, the population size parameter is set to 5, 10, 15, 20, 25, 30, 35, 40, 45, and 50. By comparing the fitness function values, the optimization effects of these 10 parameter groups are first evaluated. Then, the optimal N value is selected as a candidate solution, and fitness function values are calculated in batches (with an interval of 1) near this value, ultimately yielding the optimal parameter.

2、Maximum Number of Iterations

The maximum number of iterations must match the convergence characteristics of the algorithm: it needs to ensure the algorithm has sufficient time to approach the optimal solution, while avoiding efficiency waste caused by excessive iterations. The convergence mechanism of SMA involves global exploration in the early stage and local exploitation in the later stage, so the number of iterations must cover this complete process. In practice, the setting of the maximum number of iterations mainly affects computational efficiency. This study adheres to the principle of first ensuring the completion of SMA iterations, then appropriately reducing the number of iterations to shorten computation time. The number of iterations is set starting from 10, with an interval of 5, resulting in 10 groups: 10, 15, 20, 25, 30, 35, 40, 45, 50 (note: duplicate values in the original parameter list have been adjusted for logical consistency). Starting calculations from 10, our experimental results show that when the maximum number of iterations is set to 20, the SMA optimization algorithm has already met the iteration termination condition—thus, there is no need to test subsequent candidate parameters. Therefore, setting the maximum number of iterations to 20 can ensure the completion of SMA iterations while minimizing computation time as much as possible.

This revision fully aligns with academic paper standards: verbal examples are replaced with formula derivation, resolving the ambiguity of the original process and meeting the rigor requirements of academic research. We kindly request your re-review; if further formula details for any step need to be supplemented, we will continue to refine the content.

**Question 3:**

It is recommended to supplement comparative experiments with other optimization algorithms (such as Arctic Puffin Optimization, Particle Swarm Optimization, etc.) to verify the superiority of SMA in this context.

**Reply to Question 3:**

Thank you sincerely for your valuable comments! The issue of "completeness of the algorithm comparison system" you raised is of great significance for enhancing the persuasiveness of our research conclusions. We attach great importance to this and have completed targeted supplementary revisions as required. Here is our response regarding the specific details:

First, we would like to clarify that in the initial experimental design of this study, we had already selected three representative swarm intelligence optimization algorithms—Particle Swarm Optimization (PSO), Whale Optimization Algorithm (WOA), and Grey Wolf Optimizer (GWO)—to build a comparative reference system for the VMD hyperparameter optimization task. Through a unified experimental environment and evaluation criteria, we systematically analyzed the performance advantages of SMA in terms of optimization speed and accuracy. The relevant results have been presented in Section 4.3 of the original paper, as shown in Figures 4 and 5.

In addition, in response to your suggestion to supplement the comparison with the Arctic Fox Optimization (APO) algorithm, we have officially incorporated it into the comparison system and completed full-process supplementary verification: Under the identical experimental environment (same objective function, number of iterations, and parameter range) as the original algorithms, we tracked and recorded the optimization process of APO, obtained complete data on how its objective function value changes with the number of iterations, and integrated this data into the iteration curve of Figure 5 in the original paper. Meanwhile, in the data analysis section, we added a horizontal comparison between APO, SMA, PSO, WOA, and GWO. From the perspectives of optimization speed and accuracy, we quantitatively demonstrated the performance advantages of SMA over APO: although APO outperforms PSO, WOA, and GWO in the initial optimization stage, its convergence rate and final accuracy are still inferior to SMA. This further confirms the superiority of SMA in the VMD hyperparameter optimization task.

This revision does not alter the original research conclusions. We only enhanced the comprehensiveness and timeliness of the comparative reference system by adding a comparative experiment with the APO algorithm, which makes the performance advantages of SMA more convincing. The revised relevant figure (Figure 5) and data analysis paragraphs have been highlighted in red for your quick review. Thank you again for your rigorous evaluation and professional guidance; the revised research content is more polished, and we kindly request your further review!

**Question 4:**

It is suggested to add comparative analysis of different model combinations (such as using only Elman or TCN) in the experimental section to more intuitively demonstrate the advantages of the multi-model ensemble.

**Reply to Question 4:**

Dear Reviewer, We greatly appreciate your valuable suggestion of “supplementing comparative analysis of different model combinations”. This suggestion directly addresses the need for intuitive demonstration of the advantages of multi-model integration, and provides a critical direction for further highlighting the technical value of our study’s “time-varying multi-model integration framework”. In fact, the two-tiered demonstration framework (single-model comparison + ablation experiments) adopted in our original manuscript already covers the core model combinations you highlighted, including “using only the Elman or TCN model” and “integrating the TCN and Elman models”. To respond to your guidance, we have further supplemented the analysis of how the dynamic weighting mechanism impacts integration performance. Below is a systematic overview of the complete demonstration logic and optimized content.

In Section 4.4 of the original manuscript (“Comparison of Model Prediction Performance”), we selected the “single TCN model” as a key comparison object, aiming to verify the advantages of multi-model integration over single long-term feature models. Based on Dataset 1 (with consistent data preprocessing, training parameters, and evaluation metrics), the results show that: the single TCN model achieves an RMSE of 52.9109, MAE of 45.8468, and R² of 73.00%; in contrast, our “improved VMD + Elman-TCN integration model” reduces the RMSE to 17.2275, MAE to 15.0023, and increases the R² to 95.91%.

This result directly demonstrates that relying solely on the single TCN model fails to capture the short-term high-frequency fluctuations of DTR, while the Elman+TCN integration framework enables feature complementarity, resulting in performance significantly superior to that of the single model.

Furthermore, to further clarify the performance discrepancies among different model combinations, we designed ablation experiments in Section 4.6. Taking our proposed model (improved VMD + Elman-TCN + dynamic weighting) as the baseline, we sequentially removed key modules to generate multiple comparative models—this fully covers the model combination types you focused on:

For the single Elman model (used in isolation): It fails to capture long-term periodic features, resulting in an RMSE of 60.5068 and an R² of only 67.96%.

For the single TCN model (used in isolation): It struggles to handle short-term abrupt changes, yielding an RMSE of 52.9109 and an R² of 73.00%.Both models exhibit poor performance, as they cannot capture the dual features of DTR data (short-term high-frequency fluctuations and long-term multi-scale periodicity).

For the simple TCN/Elman integration: While it outperforms single models (RMSE reduced to 36.9609, R² increased to 81.90%), it still cannot handle extreme scenarios (e.g., coupling of strong summer radiation and gusts)—this is because it does not mitigate data non-stationarity and relies on fixed weights that cannot adapt to dynamic feature changes.

For the VMD-TCN/Elman integration (incorporating VMD decomposition): Though it resolves the non-stationarity issue (RMSE reduced to 29.0419, R² increased to 89.64%), its performance still lags behind our proposed model.

This series of gradient experiments clearly demonstrates the performance progression (from single model → simple integration → optimized integration), fully verifying that the advantages of multi-model integration do not stem from the mere superposition of models.

To further highlight the core innovative value of multi-model integration, we have added an *“Analysis of the Impact of Dynamic Weighting Mechanism”* section following the ablation experiments in Section 4.6 of the original manuscript. This section addresses the key challenge of weight allocation in multi-model integration by comparing the performance differences between *“dynamic weighting based on grey relational coefficient”* and *“traditional static weighting”*.

For this experiment, we adopted the “VMD + Elman-TCN” framework as the base architecture, varying only the weight allocation method to maintain control variables: Group 1 employed traditional static weighting, while Group 2 utilized the dynamic weighting proposed in this study. To ensure fair comparison, the experiment retained the original dataset, training parameters, and evaluation metrics. The newly added content has been fully integrated into the *“Ablation Experiments”* section (Section 4.6) of the original manuscript, and the added content has been highlighted in red for easy identification.

In summary, our revision approach follows this structure: single TCN model comparison → ablation experiments → newly added analysis of the dynamic weighting mechanism. Through this three-tiered demonstration, we have established a comprehensive comparative framework, which clearly validates the advantages of the multi-model integration architecture proposed in this study. We sincerely appreciate your professional guidance once again—this supplement has refined our demonstration system, and we have completed the revisions to the original manuscript as outlined above. We look forward to your further review.

**Question 5:**

It is recommended to explore the impact of the dynamic weighting mechanism (based on grey relational coefficients) on prediction results and validate its necessity through ablation experiments.

**Reply to Question 5:**

Dear Reviewer, We sincerely appreciate your valuable comments on our manuscript. Your suggestion — “to explore the impact of the dynamic weighting mechanism based on the grey relational coefficient on prediction results and verify its necessity via ablation experiments” — directly aligns with the core logic of integrated model optimization, and it is instrumental in enhancing the persuasiveness of our work. Following your feedback, we have completed targeted revisions, and we outline the specific changes and relevant explanations below:

The dynamic weighting mechanism is a critical component of the time-varying multi-model integration framework proposed in this study. Its core value lies in addressing the limitation of insufficient prediction accuracy in traditional fixed-weight integrated models. To validate the effectiveness of this mechanism, we have added a dedicated control group for ablation experiments: by comparing models with and without the dynamic weighting mechanism, we quantitatively analyze its contribution to improving prediction performance and clarify its irreplaceable role in the integrated model.

In Section 4.5 (“Ablation Experiments”) of the original manuscript, we have added a new control group for ablation experiments. The model architecture of this control group is: Improved VMD-Elman/TCN Integrated Model with Fixed Weighting Mechanism. Specifically, this control group shares the same base architecture as our proposed model, except that the grey relational coefficient-based dynamic weighting mechanism is replaced with a traditional fixed-weight allocation strategy (adopting the commonly used equal-weight scheme in the field, where both the Elman model and TCN are assigned a fixed weight of 0.5). To ensure rigor, the new control model shares identical training datasets, hyperparameter configurations, and evaluation metrics with the original model; the only variable is the weight allocation mechanism, allowing us to precisely isolate the impact of the dynamic weighting mechanism.

The results of this new experiment can be found in the highlighted (red) section of Section 4.5 (“Ablation Experiments”) in the revised manuscript.

We would like to express our sincere gratitude again for your rigorous guidance. Through this targeted additional ablation experiment, we have fully validated the core role of the grey relational coefficient-based dynamic weighting mechanism in enhancing prediction accuracy and generalization capability, as well as its irreplaceability in the time-varying multi-model integration framework. The revised manuscript has been further enhanced in terms of logical completeness and scientific rigor of the demonstration. We have revised the entire manuscript in accordance with the journal’s formatting requirements, and we kindly request your further review.

**Question 6:**

It is suggested to add detailed analysis of different datasets in the experimental results to demonstrate the adaptability and stability of the model under various scenarios.

**Reply to Question 6:**

We sincerely appreciate the valuable feedback you provided. Your suggestion to “incorporate detailed analysis of different datasets in the experimental results to demonstrate the model’s adaptability and stability across various scenarios” directly addresses the validation of the study’s engineering practical value; we take this seriously and have completed targeted supplementary revisions. We outline the details of these adjustments below:

Your focus on “detailed analysis of different datasets” has been addressed centrally in Section 4.6 of the original manuscript (“Model Generalization Verification Experiments”). We have added a dedicated ~250-word analysis section following the existing experimental result table and basic analysis (this section has been highlighted in red in the revised manuscript). Additionally, we have supplemented the quantitative description of scenario differences between datasets in Section 4.6, establishing a complete logical chain: data characteristics → experimental performance → model adaptation mechanism — which fully demonstrates the model’s adaptability and stability.

The core of these revisions is as follows: By comparing the scenario differences (meteorological fluctuation intensity, geomorphic environment, and DTR data characteristics) between the two datasets, we conduct an in-depth analysis of the model’s performance and adaptation principles across distinct scenarios. For example:

- For Dataset 1 (summer, industrial area, high-frequency fluctuations), we analyze how the model achieves low-error prediction via SMA-VMD (to decompose non-stationary data) and Elman (to capture short-term abrupt changes);

- For Dataset 2 (late winter to early spring, suburban area, stable trends), we explain how TCN’s long-term feature extraction and the dynamic weighting mechanism ensure high fitting accuracy.

Additionally, by quantifying the fluctuation range of the model’s performance across the two datasets, we directly verify the model’s stability.

This revision does not alter the original experimental conclusions; instead, it strengthens the demonstration of the model’s adaptability and stability through in-depth analysis of scenario differences → performance correlation → mechanism explanation, making the experimental results more convincing. We kindly request your further review.

**Question 7:**

It is recommended to supplement the sensitivity analysis of model prediction results to explore the impact of different parameter settings on prediction outcomes.

**Reply to Question 7:**

Dear Reviewer, We sincerely appreciate your valuable suggestion to “supplement sensitivity analysis of the model’s prediction results”. This suggestion directly targets the core validation logic for the scientific rigor of parameter selection: by analyzing how changes in key parameters impact prediction outcomes, we can not only further justify the rationality of our current parameter configuration but also clarify the model’s adaptability across different parameter ranges — thereby enhancing the completeness of our experimental framework. Following your guidance, we have conducted a systematic sensitivity analysis on the model’s core parameters (key parameters of the SMA algorithm, VMD hyperparameters, and dynamic weighting sliding window length). We outline the specific analysis logic and results below:

This sensitivity analysis focuses on core parameters that directly affect prediction accuracy. We adopted a single-variable control approach: fixing all parameters except the target one at their current optimal values, then observing the fluctuation patterns of prediction metrics as the target parameter varies within a reasonable range. This process ultimately verifies the scientific validity of our parameter selection.

First, for the core parameters of the SMA algorithm (population size, maximum number of iterations):

We adjusted the population size from 10 to 25 (step size = 5) and the maximum number of iterations from 10 to 50 (step size = 10), analyzing their impacts on the optimization performance of VMD hyperparameters and the final DTR prediction accuracy. The results show that:

When the population size is 10 or 15, the predicted R² stabilizes around 95.4%. At a population size of 20, the R² stabilizes at 95.9%; if the size is less than 15, R² drops below 94%; if greater than 20, no significant accuracy improvement is observed, but computational time increases—validating the rationality of selecting a population size of 20.

When the maximum number of iterations is 10, the predicted R² stabilizes around 95.9%; as the number increases to 20, 30, 40, and 50, accuracy remains unchanged while computational time rises gradually. Note that using 20 iterations (to align with the SMA vs. other optimization algorithms comparison) may prevent some algorithms from meeting their termination conditions; thus, we retain 20 as the maximum iteration count in Section 4.3 of the manuscript.

Second, for the key VMD hyperparameters (number of decomposition components *K*, penalty factor α):

We adjusted *K* from 3 to 12 (step size = 1) and α from 200 to 2600 (step size = 500), observing their impacts on DTR data decomposition performance and subsequent prediction. The results indicate that:

When *K*, the envelope entropy of decomposed components is minimized, and the predicted R² reaches 95.91%. For K<8, mode mixing occurs in components, leading to varying degrees of R² reduction; for K>10, over-decomposition of DTR data increases redundant components, reducing R² and extending computational time—confirming *K* = 9 as optimal.

When α < 1200, partial spectral overlap exists in decomposed components, causing inconsistent R² values (without reaching the optimal level); when α > 1200, overlap rate rises continuously, and R² decreases steadily—validating the rationality of α = 1200.

Third, regarding the sliding window length for dynamic weighting: We adjusted the window length from 5 to 35 (with a step size of 5) and analyzed its impacts on the timeliness of weight calculation and prediction accuracy. The results demonstrate that when the window length is less than 20, insufficient historical data leads to significant fluctuations in weighting coefficients, with the model’s coefficient of determination (R²) peaking at only 84.32%. When the window length is set to 25, the weighting coefficients can well adapt to the variations in dynamic thermal rating (DTR) data in real time, and the prediction R² remains stable within the range of 92.93%–95.91%. For window lengths greater than 25, weight updates lag behind the actual data changes; although the prediction R² stabilizes at approximately 94.50% without further improvement, the associated computational time increases considerably. Therefore, a window length of 25 was selected for this study.

In summary, the above sensitivity analysis has clarified the influence patterns of core parameters on the prediction outcomes: the model achieves optimal accuracy under the current parameter configuration and exhibits favorable robustness within a reasonable parameter range. We have supplemented the aforementioned analysis to the corresponding experimental section “4.7” of the original manuscript, which strengthens the scientific justification for the selection of model parameters. We respectfully request that you conduct a re-review of this revised content.

**Question 8:**

It is suggested to further discuss the application prospects and potential challenges of this method in practical engineering in the discussion section, such as deployment costs and real-time requirements in large-scale power grids.

**Reply to Question 8:**

We sincerely appreciate your attention to the engineering application value of this study and your constructive suggestions. Your proposal to further explore the application prospects and potential challenges of the method in practical engineering (e.g., large-scale power grid deployment costs, real-time performance requirements) is of great significance for enhancing the practicality and completeness of our research. We have carefully revised Section 5 ("Conclusions") of the original manuscript in targeted response to your request, and we now detail the modifications as follows:

First, we systematically organized the core conclusions of the method by integrating the experimental validation data from the original manuscript with the actual operating conditions of electrical engineering. Building on this, we candidly analyzed the inherent limitations of the method during its large-scale implementation—grounded in the method’s principles and practical electrical engineering application scenarios—to avoid overgeneralized exaggeration of its capabilities:

**1、Data dependence in complex scenarios**: The high-precision prediction of the method relies on high-quality micro-meteorological data (e.g., wind speed, ambient temperature) sampled at 15-minute intervals. For regions with sparse meteorological monitoring stations (e.g., remote mountainous areas, high-altitude zones), if micro-meteorological monitoring equipment for key transmission towers cannot be supplemented, missing or low-precision data will degrade prediction performance. This constitutes the core constraint for the method’s large-scale promotion.

**2、Lightweight requirements for ultra-large-scale power grids**: The method comprises three core modules: SMA-VMD decomposition, dual-model prediction, and dynamic weighting. While the prediction latency for a single line is relatively low, the overall computing power consumption during multi-node parallel computing remains suboptimal for ultra-large-scale power grids (with thousands of lines). The computing power redundancy of existing edge computing nodes may fail to meet real-time scheduling demands under extreme conditions.

Finally, we have supplemented the summary and future optimization directions in detail. At the end of the section, we explicitly conclude: The method proposed herein has clear application value for dynamic capacity expansion of regional power grids with complete meteorological data and complex climatic conditions. However, constrained by data dependence and computing power requirements, its large-scale engineering deployment must be advanced in phases aligned with regional monitoring conditions. Future research will focus on model lightweight optimization and robustness improvement in data-scarce scenarios, to further expand the boundaries of its engineering applications.

The above summarizes our modifications. If you wish to review the full detailed changes, please refer to the red-highlighted text in Section 5 ("Conclusions") of the original manuscript.

**Question 9:**

It is recommended to add a more comprehensive review of existing research in the introduction section to highlight the position and contribution of this study in the existing literature. The literature review could benefit from citing the following recent works to provide a broader perspective. Such as: https://doi.org/10.1007/s12145-025-01966-y; https://doi.org/10.1016/j.jhydrol.2025.134304.

**Reply to Question 9:**

We sincerely appreciate the valuable feedback you provided! Your suggestion to supplement a comprehensive literature review and clarify the positioning and contributions of this study within the existing literature is highly instructive. We attach great importance to this comment and have completed targeted revisions as requested. We now detail the modifications below:

We structured this revision around the logical framework of “cross-domain technology reference – scenario adaptation limitations – innovative breakthroughs of this study”. We incorporated the two high-quality studies in the runoff prediction field (DOI: 10.1007/s12145-025-01966-y and 10.1016/j.jhydrol.2025.134304) into the literature review system of the DTR prediction domain: we acknowledge the reference value of their technical innovations for time-series prediction tasks, while clarifying their adaptation limitations in the specific DTR scenario. Ultimately, we anchored the discussion to the core innovations of our study, enhancing the continuity and targeting of our research.

The revisions are specifically implemented in the transitional paragraph of Chapter 0 (Introduction) of the original manuscript, between the sections on “Limitations of single models” and “Handling non-stationarity of DTR data”. We added approximately 300 words of literature review content in this section, and the revised paragraph in the original manuscript has been highlighted in red for your convenient and quick reference. We again thank you for your rigorous review and valuable guidance; the revised introduction can better support the academic value and application positioning of our study. We kindly request your further review of the revised content!

**Question 10:**

There is inconsistent use of terminology and symbols in the method description and experimental sections, which should be standardized for consistency.

**Reply to Question 10:**

Dear Reviewer, We sincerely appreciate your valuable comment regarding the inconsistent use of terminology and symbols in the method description and experimental section, as well as the need for standardized unification. We attach great importance to this issue and promptly conducted a section-by-section cross-checking and self-audit of these sections throughout the manuscript. We identified typical inconsistencies and have addressed them comprehensively. Below is a detailed response:

Through cross-chapter verification, we found that the terminology and symbol inconsistencies primarily fall into two categories: (1) non-standard formatting of evaluation index symbols and numerical symbols; (2) synonymous confusion in the naming of technical methods. While these issues did not compromise the core conclusions, they reduced the manuscript’s readability and professionalism. The specific issues and corrections are as follows:

1、 **Non-standard formatting of evaluation index/numerical symbols**

Per academic conventions, indices such as Root Mean Square Error (RMSE) and coefficient of determination (R²) require triple consistency: symbol formatting, terminology description, and unit labeling. Our self-audit revealed:

In Section 2.6 (method description), the RMSE symbol was not italicized; however, it appeared in italics in the table of Section 4.4 (experiments).

Per the manuscript’s symbol standards: variable symbols should be italicized, while constants and meaning-specific subscripts use roman font. The model weight coefficient “w” was unitalicized in the method section but italicized in the experimental section.

To resolve this, we standardized formatting uniformly: all evaluation index symbols are presented in italics.

2、 **Synonymous confusion in core technical method naming**

The core innovation (“weight allocation mechanism based on grey relational coefficient”) was termed “dynamic weighting mechanism” in Section 3.5 (method description) but incorrectly referred to as “adaptive weighting strategy” in the new analysis of Section 4.6.

“SMA-optimized VMD algorithm” (defined in Section 2.4 as the improved VMD decomposition) was simplified to “optimized VMD” in the experimental section, causing inconsistent references.

We have standardized the naming of core methods: a unique name is specified in the method section, and we explicitly note that this abbreviation will be strictly used in all subsequent chapters (from its first definition) to ensure consistent reference.

We again thank you for your professional guidance—this standardization of terminology and symbols has enhanced the manuscript’s academic rigor. We have attached the fully revised manuscript, with all modifications highlighted in red for your efficient review.
